# Macro- and Nanoscale Effect of Ethanol on Bovine Serum Albumin Gelation and Naproxen Release

**DOI:** 10.3390/ijms23137352

**Published:** 2022-07-01

**Authors:** Niuosha Sanaeifar, Karsten Mäder, Dariush Hinderberger

**Affiliations:** 1 Institute of Chemistry, Martin Luther University Halle-Wittenberg, Von-Danckelmann-Platz 4, 06120 Halle (Saale), Germany; niuosha.sanaeifar@chemie.uni-halle.de; 2 Institute of Pharmacy, Martin Luther University Halle-Wittenberg, Wolfgang-Langenbeck-Str.4, 06120 Halle (Saale), Germany; karsten.maeder@pharmazie.uni-halle.de

**Keywords:** albumin, hydrogels, ethanol, EPR spectroscopy, release behavior

## Abstract

We report extended ethanol-induced gelation procedures of bovine serum albumin (BSA) at 37 °C and investigate the release behavior of a spin-labeled naproxen derivative (SL-NPX) from these hydrogels. The macroscopic mechanical properties of these gels during formation were studied using rheology, while a nanoscopic, more molecular view was obtained by analyzing the secondary structure of the protein during gelation via infrared (ATR-IR) spectroscopy. To evaluate the potential use of BSA hydrogels in controlled drug delivery, SL-NPX-BSA interaction was investigated in detail by continuous-wave electron paramagnetic resonance (CW EPR) spectroscopy, which provides information on the interaction of the small drug molecules and the hydrogel. In addition to CW EPR spectroscopy, dynamic light scattering (DLS), which provides insight into the size and nature of released components, was applied to characterize the combined influence of incubation time, ethanol, SL-drug, and BSA concentration on release behavior. It was found that the alteration of initial drug loading percentage, hydrogel incubation time as well as BSA and alcohol concentrations affect and thus tune the release rate of SL-NPX from BSA hydrogels. These results lead to the conclusion that BSA hydrogels as controlled release systems offer a remarkable fine-tuning capability for pharmaceutical applications due to the variety of gelation parameters.

## 1. Introduction

Many research studies have contributed to the design and development of proper targeted dosage forms to treat diseases and improve patient compliance [1]. Some of the drugs have a short lifetime and low efficacy due to nonspecific biodistribution and rapid consumption by the body, therefore they require frequent administration at high doses to achieve the desired plasma level and therapeutic activity [2,3]. However, repetitive dosing can cause several problems, such as high toxicity, low therapeutic efficiency, and severe side effects on tissues [4]. To counteract these deficiencies, controlled and sustained drug delivery systems, providing a constant and prolonged drug concentration, have been developed and have attracted significant attention [5]. Several systems, among them nanofibers [6,7], microparticles [8,9,10], smart polymers [11,12], hydrogels [13,14,15], nanocarriers [16], etc., have been explored for drug delivery applications.

Hydrophilic hydrogels with a three-dimensional polymeric network synthesized by chemical or physical crosslinking are capable of retaining and stabilizing a large volume of water or biological fluids [17]. The presence of polar functional groups results in the extensive water-swollen content, while resistance to dissolution arises from crosslink density [18]. Several types of hydrogels have emerged as a promising option for various pharmaceuticals and medical applications such as drug release, wound dressing, and tissue engineering due to their biocompatibility, biodegradability, high porosity, and flexibility [19,20].

**Figure 1:**
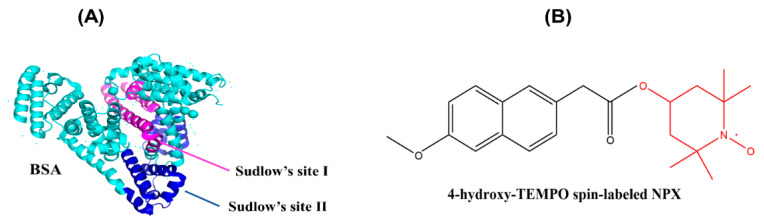
(**A**) Three-dimensional structure of BSA and its drug-binding sites (the protein topology from PDB ID 4f5s) and (**B**) chemical structure of SL-NPX.

Protein-based biomaterials are widely used in tissue engineering, regenerative medicine, and drug delivery applications. We work with bovine serum albumin (see Figure 1A), which can act as a drug carrier and provide controlled delivery of therapeutic agents [21]. In addition, due to its unique properties such as biodegradability, biocompatibility, high stability, and low toxicity, BSA is selected as a protein model in numerous research studies [17]. Generally, albumin is the major plasma protein performing crucial roles in the maintenance of osmotic pressure and blood pH and transport of various ligands such as insoluble fatty acids (FA), metal ions, hormones, and a great number of drug compounds due to the presence of different binding sites [18,22].

Albumin hydrogel formation by thermally and pH-induced methods has previously been well established [23,24]. Heat-induced gelation involves applying high heat which leads to the denaturation of albumin and unfolding of its structure at temperatures above 62 °C [25]. This leads to the availability of functional groups engaged in intramolecular bonding for intermolecular interactions. Hydrophobic interactions due to the exposure of hydrophobic regions lead to protein aggregation and the final gel network [26]. In the pH-induced method, albumin transits from the N-form to the F-form isomer by lowering the pH to 3.5 resulting in the hydrophobic interactions and subsequent gelation [18]. Our previous work has shown hydrogel formation from bovine serum albumin below its denaturation temperature at 59 °C [14]. Furthermore, we have recently investigated gel formation at 37 °C by the addition of different ethanol concentrations as chemical denaturants to BSA precursor solutions [27].

Only in recent years, has serum albumin been extensively studied as a drug delivery carrier for controlled drug release. For instance, Upadhyay et al. synthesized BSA hydrogel using epichlorohydrin as a self-healing and injectable material to study the release of doxorubicin [28]. Iemma et al. prepared pH-sensitive bovine serum albumin microspheres to investigate release profiles of diflunisal and β-propranolol [29]. One experiment carried out by Hirose et al. elucidated the formation of a hydrogel consisting of recombinant human serum albumin as a physiological carrier for sodium benzoate, salicylic acid, and warfarin release [30].

Naproxen (NPX), a nonsteroidal anti-inflammatory drug, is commonly used to relieve fever and to treat osteoarthritis, rheumatoid arthritis, metastatic bone pain, inflammation, headaches, and migraines. Due to the short bioavailability of the drug (8 h) by oral administration, frequent dosing is required to maintain pharmacological action. However, repeated administration in patients with chronic inflammatory disorders which require long treatment with naproxen can result in gastrointestinal side effects like bleeding. For this reason, it seems crucial to develop a controlled drug delivery system to reduce the frequency of administration and allow the sustained release of drug. Moreover, the development of a system for local release and treatment could be a viable application of albumin-based hydrogels [31,32,33]. Previously, our group synthesized a wide variety of spin-labeled pharmaceuticals (SLP) including naproxen (see Figure 1B) as ligands and studied ligand uptake by human serum albumin in serum-like concentration solution through continuous-wave EPR spectroscopy [34].

The present investigation was directed towards the development of a controlled drug delivery system that may be applied in local delivery based on BSA hydrogels that were prepared by mixing different amounts of ethanol and BSA and incubation at 37 °C and studying the release behavior of SL-NPX from the prepared gel. In previous studies, the release behavior of 16-doxyl stearic acid (16-DSA) as a model drug, spin-labeled warfarin, and coumarin-3-carboxylic acid from BSA hydrogels, prepared by thermally and pH-induced methods, and nanoscopic properties of small molecule–hydrogel interactions was analyzed in depth [14,15]. The objective of the present contribution is to investigate the potential and the effect of ethanol concentration on hydrogel formation as well as the influence of parameters such as incubation time (time required for gel formation), BSA, ethanol, and SL-NPX concentrations on release rate. Since ethanol as a gelating stimulus presents a disadvantage for parenteral application in comparison to heat, in particular, use as a local delivery system might be envisioned rather than one for systemic release. We thus characterize mechanical properties as well as secondary structure changes of prepared gels using rheology and ATR-IR spectroscopy, analyze the combined effects of mentioned parameters and NPX-BSA interaction by means of CW EPR spectroscopy, and study the size and nature of released components by DLS.

## 2. Results and Discussion

### 2.1. Measurement of Rheological Properties

The exact tuning of mechanical and viscoelastic properties of hydrogel biomaterials is crucial for tailoring hydrogels for certain pharmaceutical and biomedical applications. For instance, in the field of tissue engineering, the toughness of the gel is an essential factor for keeping a sustained scaffold to regulate cell behavior, while in pharmaceutical applications, a hydrogel must have a proper flowability to be an excellent candidate to act as an injectable drug delivery carrier [14,35].

The magnitude, number, and pattern of intermolecular interactions including hydrophobic, solvation, and van der Waals, which facilitate protein network formation, determine the rheological properties of protein solutions and hydrogels [23]. In order to analyze the effect of ethanol concentrations on BSA gel formation at 37 °C, time-dependent rheological measurements were performed on the rheometer plate. It is important to note that the total volume of each sample was 2000 µL; therefore, the appropriate amounts of BSA and ethanol were added and filled up with water to achieve the final volumes and concentrations.

**Figure 2:**
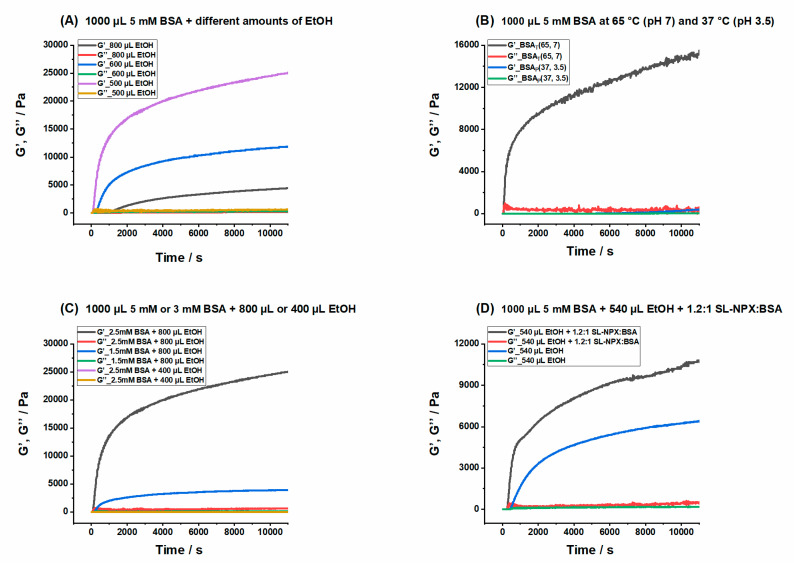
Time-dependent elastic (G′) and viscous moduli (G′′) of (**A**) 1000 µL 2.5 mM BSA_E_(37, 7) with different amounts of ethanol, (**B**) 1000 µL 2.5 mM BSA_T_(65, 7) and BSA_P_(37, 3.5), (**C**) 1000 µL 2.5 mM or 1.5 mM BSA_E_(37, 7) with 800 µL or 400 µL ethanol and (**D**) 1000 µL 2.5 mM BSA_E_(37, 7) with 540 µL ethanol in the presence and absence of SL-NPX. Total volume of each sample is 2000 µL, which is reached by adding appropriate amounts of water to the mixture of BSA, ethanol, and SL-NPX to reach the desired final volume.

Figure 2A shows the storage (G′) and loss (G′′) moduli curves of 1000 µL 5 mM BSA precursor solution with three different amounts of ethanol at pH 7 and 37 °C (see Figure S1 for mechanical properties of 1000 µL 3 mM BSA with different ethanol concentrations). In the sample with 800 µL ethanol, the storage modulus surpassed the loss modulus instantly, indicating immediate gel formation. The G′ value of this hydrogel is around 23,000 Pa after 2 h. According to our definition for mechanically robust hydrogels, the storage modulus should exceed 10,000 Pa after 1 h. Therefore, tough hydrogels can be produced with higher volumes of ethanol. However, the visual assessment showed that much higher amounts of ethanol lead to the formation of large white BSA aggregates, immediately after the addition of ethanol to the BSA precursor solution. High alcohol concentrations cause intense and very fast denaturation, with partial protein network formation, finally resulting in aggregate formation [36,37]. As can be expected, the secondary structure of BSA is significantly affected by the addition of ethanol, as is extensively discussed in the IR section below. The gelation points in hydrogels with 600 µL and 500 µL ethanol were reached after 3 and 10 min, respectively, and their final/plateau G′ values were considerably lower (≈11,000 and 3500 Pa, respectively) than that of the gel with 800 µL ethanol. It is important to note that much larger incubation times are required for gel formation at lower ethanol volumes (below 400 µL) and the final gel is considered to be extremely weak.

The results of ethanol-induced hydrogels are compared with gels prepared by the other methods to gain insight into the mechanical properties of these hydrogels. Figure 2B shows the BSA hydrogels prepared by thermally and pH-induced methods. In our previous work, BSA hydrogels generated slightly above and slightly below the denaturation temperature (at 65 °C and 59 °C) at pH 7, and by lowering the pH to 3.5 at 37 °C, have been extensively investigated [14,15]. We obtain thermally induced hydrogels by heating the precursor solution of 1000 µL, 5 mM BSA above the denaturation temperature of this protein at 65 °C. The gelation starts instantly and the G′ value reaches 13,000 Pa after 2 h. However, gel formation in pH-induced hydrogels has a relatively slow formation rate, with a delayed onset of gelation after 40 min and a G′ value of only around 75 Pa after 2 h, indicating a very weak gel network. By comparing Figure 2A,B, we can conclude that it is possible to form hydrogels with different mechanical properties by tuning the ethanol concentrations. Moreover, the addition of high volumes of ethanol allows us to obtain hydrogels at 37 °C and neutral pH that are mechanically as robust as those formed by increasing the temperature to 59/65°C and lowering the pH to 3.5.

In Figure 2C, we also present the correlation between BSA concentration and ethanol concentration. The data clearly show that increasing the amount of ethanol leads to a higher G′ value, and a much more robust hydrogel is formed in spite of the low protein concentration. Moreover, gelation sets in instantly when high amounts/volumes of ethanol are used, while by the addition of only 400 µL ethanol, no gel is formed even after 3 h. However, by keeping the amount of ethanol constant, it can be clearly seen that a higher BSA concentration leads to more mechanically tough hydrogels.

The impact of SL-NPX on ethanol-induced gelation is investigated by the addition of this SL-drug to the precursor solution of BSA and rheological analysis was repeated at 37 °C. It seems that the addition of SL-NPX results in a higher storage modulus compared to the BSA hydrogels prepared without SL-NPX. Apparently, SL-NPX can facilitate gel formation and enhance mechanical properties. As is elucidated in the CW EPR results, ethanol-induced gelation results in a lower binding capacity; therefore, SL-NPX may interact with the surface of the protein instead of binding to Sudlow sites of BSA, which can enhance denaturation and faster gelation onset. We have previously studied the effect of SL-coumarin-3-carboxylic acid and SL-warfarin on gel formation by thermally and pH-induced methods. The SL-drugs could weaken the mechanical properties of temperature-induced hydrogels; however, the G′ value increased slightly when hydrogels were generated by reducing pH [15].

### 2.2. ATR-IR Spectra of Ethanol-Induced BSA Hydrogels

**Figure 3:**
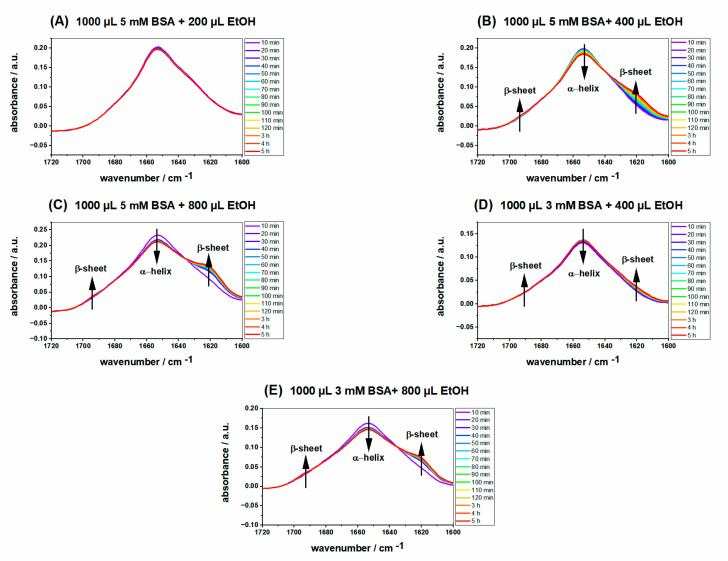
Time dependence of IR absorption spectra of 1000 µL (**A**) 5 mM BSA precursor solution with 200 µL ethanol, (**B**) 5 mM BSA precursor solution with 400 µL ethanol, (**C**) 5 mM BSA precursor solution with 800 µL ethanol, (**D**) 3 mM BSA precursor solution with 400 µL ethanol, and (**E**) 3 mM BSA precursor solution with 800 µL ethanol. All of the samples were measured at 37 °C on ATR IR crystal.

The secondary structure of native BSA consists of 67% α-helices, 10% β-turn, and 23% extended chain, without any β-sheets. It is well known that the secondary and tertiary structures of BSA can be affected by physical and chemical factors such as pH, temperature, and the addition of various kinds of denaturants [38]. For instance, β-sheet content increases by heating, while no β-sheet is contained in native BSA. It has been shown that BSA gelation occurs through the formation of intermolecular β-sheets and disruption of α-helical structures [39]. To explore the ethanol-induced changes in the secondary structure of BSA at 37 °C, time-dependent ATR IR measurements were carried out during the first five hours of gelation. The appearance of two shoulders at about 1620 and 1680 cm^−1^ indicates β-sheet structure formation, the hallmark of intermolecular aggregation of BSA and gel formation [40]. As can be seen in Figure 3A, in spite of the high BSA concentration, no significant time-dependent changes can be detected in the IR spectra due to the low amount of ethanol (200 µL), indicating that no gel is formed in this sample. In contrast, these bands rise independently of BSA concentration for all other samples with higher ethanol volumes (400 µL and 800 µL), and the peak at around 1650 cm^−1^, which is attributed to α-helices, decreases, suggesting that α-helices and intramolecular β-sheets are converted to intermolecular β-sheets, which leads to a three-dimensional network structure and gel formation. When ethanol, which is not as polar as water, is added to the precursor protein solution, the van der Waals interactions (hydrophobicity) which separate the nonpolar groups of BSA from the surrounding water, are screened, resulting in the partial unfolding of the protein [41].

For a better understanding of the hydrogel formation and structural evolution for the ethanol-induced method, the ratio of α-helix to β-sheet content was quantified and the intensities of both bands were monitored accurately [I(α-helix)/I(β-sheet)].

**Figure 4:**
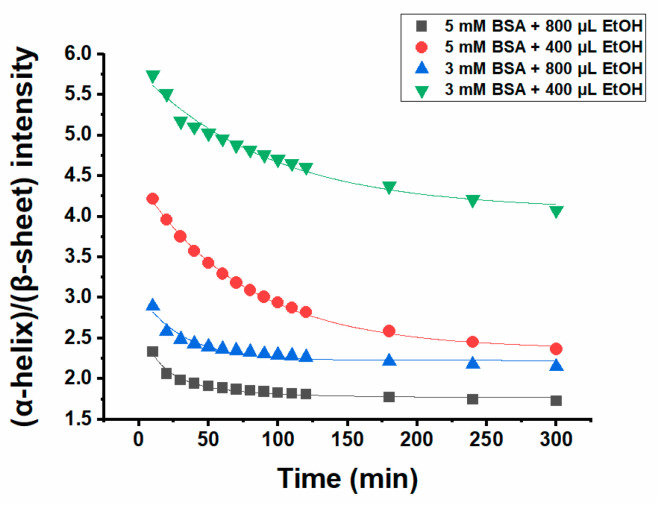
I(α-helix)/I(β-sheet) as a function of time for 1000 µL 5 mM or 3 mM BSA precursor solutions with 800 µL or 400 µL ethanol at 37 °C. The solid lines are fitted curved.

Figure 4 shows the intensity of (α-helix)/(β-sheet) during the gel formation process of 1000 µL 5 and 3 mM BSA with 400 µL and 800 µL ethanol, respectively, giving four samples altogether. Comparison of the hydrogels prepared with the same BSA concentration but a higher amount of ethanol (800 µL) with the one formed by the addition of a lower ethanol volume (400 µL) reveals that the (α-helix)/(β-sheet) ratio is much lower for the former, implying stronger conformational changes for the hydrogel with a higher ethanol concentration. Moreover, the ratio of (α-helix)/(β-sheet) for the hydrogels with the same amount of ethanol but different concentrations of BSA is higher when the hydrogel is formed by a lower BSA concentration. The results of ATR IR spectroscopy are in good agreement with the rheological measurements, as the value of the storage modulus for hydrogels prepared by higher BSA and ethanol concentrations is much higher compared to the ones made from low concentration of BSA and low ethanol content. This is an indication that there is a meaningful correlation between conformational changes, i.e., changes in the secondary structure content of the protein and its mechanical properties. In our previous work, we extensively studied the conformational changes of BSA_T_(65, 7) and BSA_P_(37, 3.5) alone and how the addition of 16-DSA affects the secondary structure of protein during gel formation [14].

### 2.3. CW EPR Spectroscopy

#### 2.3.1. Characterization of BSA Hydrogels

Two high-affinity drug binding sites have been proposed in human serum albumin (HSA), known as Sudlow site I and Sudlow site II. Sudlow site I is located in subdomain IIA and seems to preferentially bind to large heterocyclic and negatively charged compounds, while site II can bind small aromatic carboxylic acids [42,43]. It has previously been shown that HSA has two high-affinity binding sites for SL-NPX [34]. It must be mentioned that the crystal structure and binding characteristics of BSA demonstrate a high resemblance to HSA [43]; although, in the solution state, HSA seems to gain more flexibility than BSA [44,45,46]. We obtained precise data on the HSA binding affinities of various pharmaceuticals in solution by spin labeling and CW EPR spectroscopy, which provide information on protein-binding capabilities as well as the motional freedom of SL-drugs [34].

The investigation of the albumin hydrogels–FA interaction reveals strong FA-binding capacities of BSA and HSA alone as well as their hydrogels; however, the preparation method can determine the number and strength of their attachment [23]. Furthermore, by performing EPR measurements and simulations, it is possible to gain insight into the rotational motion. In its simplest, isotropic form, rotational motion is characterized by rotational correlation time τc calculated from the simulated diffusion tensor *D* (τc = 1/6(*DxxDyyDzz*)^−1/3^). This parameter is in the range of 10 ps for fast tumbling components to a few microseconds for strongly immobilized molecules. In addition, the value of the isotropic 14N-hyperfine coupling constant *a*_iso_ gives information on the polarity of the nitroxide group environment. High polarities result in larger *a*_iso_ values, while less polar environments lead to smaller hyperfine couplings. We here present the characterization of the SL-NPX-loaded hydrogels as well as the release behavior of this SL-pharmaceutical from ethanol-induced gels.

**Figure 5:**
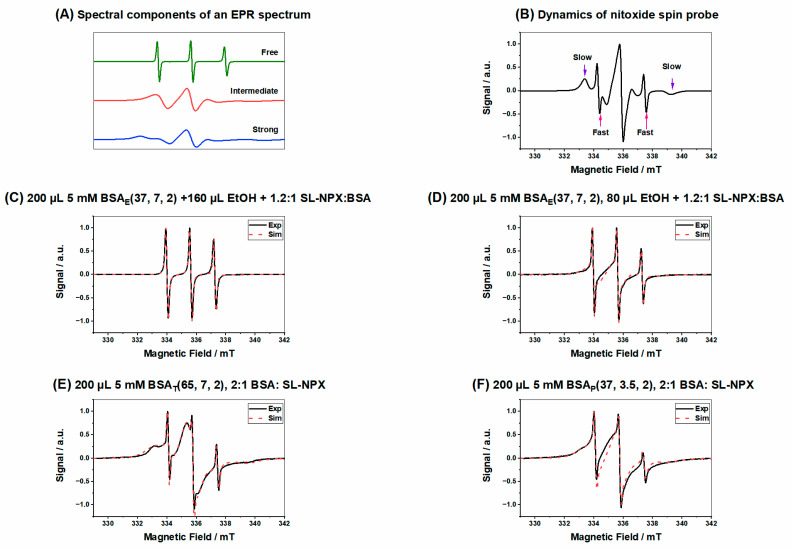
(**A**) Individual components of nitroxide radicals in EPR spectra, as derived from spectral simulation of different rotational motion, (**B**) features of the different components of an EPR spectrum for nitroxide spin probe, (**C**) SL-NPX-loaded BSA_E_(37, 7, 2) at a 1.2:1 SL-NPX:BSA molar ratio with 160 µL ethanol, (**D**) SL-NPX-loaded BSA_E_(37, 7, 2) at a 1.2:1 SL-NPX:BSA molar ratio with 80 µL ethanol, (**E**) SL-NPX-loaded BSA_T_(65, 7, 2) at a 2:1 SL-NPX:BSA molar ratio, and (**F**) SL-NPX-loaded BSA_P_(37, 3.5, 2) at a 2:1 SL-NPX:BSA molar ratio.

Figure 5A displays the analysis of the spectral composition of the SL-NPX EPR spectra. All measured spectra contain these three components in varying fractions/weights. They all show unbound, intermediately and strongly immobilized components in the EPR spectra, while different regions of the EPR spectrum for nitroxide spin probes, highlighting characteristic features of all components, are shown in Figure 5B. When plotting the different weights of the three components with time, one can follow the nanoscale environment/binding behavior of the SL-drug. EPR spectra of ethanol-induced hydrogels with the same BSA and SL-NPX amounts and two different amounts of ethanol (160 µL and 80 µL) are shown in Figure 5C,D. It is important to note that the total volume of each sample was 400 µL; therefore, the appropriate amounts of BSA, ethanol, and SL-NPX were added, and the rest was filled with water.

Spectral simulations reveal that almost 48% of SL-NPX shows freely tumbling rotation and 51% has intermediate rotational motion and there is no sign of a strongly bound component when the hydrogel was prepared with 160 µL ethanol. However, by decreasing the volume of ethanol to 80 µL, the percentage of freely rotating SL-NPX decreases to 10%, while that of the intermediately bound ligand to BSA increases to 67%. Moreover, for this hydrogel, almost 22% of SL-NPX is tightly immobilized to protein. It seems that by keeping the amounts of BSA and SL-drug constant and increasing the volume of ethanol, SL-NPX rotates much faster. In general, EPR data indicate that the presence of ethanol in the solution and hydrogels can strongly weaken the BSA alpha-helical secondary structure (the strong binding sites are in fact located at the interface of alpha helices), resulting in screened albumin SL-NPX interactions and higher amounts of free drug. Moreover, ethanol can be considered a good solvent for SL-NPX, so this SL-drug prefers ethanol solvation instead of BSA binding sites (which it would prefer in water). In order to gain an accurate and deep understanding of the ethanol-induced hydrogels, we have compared this gelation technique with other hydrogel formation methods. As can be seen in Figure 5E, in the thermally induced hydrogel (preparation at 65 °C), almost 73% of SL-NPX is tightly bound to BSA, 24% has intermediate rotational motion, and only 2% shows freely tumbling rotation. However, by the addition of HCl and reducing the pH to 3.5, the percentage of intermediately bound and freely tumbling SL-NPX increases to 31% and 4%, while that of the tightly immobilized SL-pharmaceutical decreases to 65% (Figure 5F). The main parameters gained from EPR spectral simulations shown in Figure 5 are summarized in Table 1. As explained above, BSA has two high-affinity binding sites at the interfaces of α-helices for SL-NPX which leads to tight immobilization of ligand to BSA. Comparison of the ethanol-induced hydrogels with the ones synthesized by thermally and pH-induced methods reveals that the fraction of freely tumbling SL-NPX is higher when a higher concentration of ethanol is added to the precursor solution. In other words, ethanol-induced hydrogels can lower the SL-pharmaceutical binding capacity resulting in weaker interactions between BSA and SL-NPX in comparison with other gel preparation methods. It is important to note that all of the studied hydrogels were prepared by keeping the samples in a thermomixer for 2 h. The effect of ethanol concentration on the binding capacity of FAs in BSA hydrogels has been described and analyzed in detail before [27]. The results of EPR measurements are comparable to the rheological and ATR IR characterizations, as high ethanol concentrations have a significant denaturation effect on BSA, which leads to mechanically robust hydrogels at the cost of reducing the number of classical binding sites. Further information about the potential effects of other ethanol concentrations on gelation can be found in the Supporting Information.

**Figure 6:**
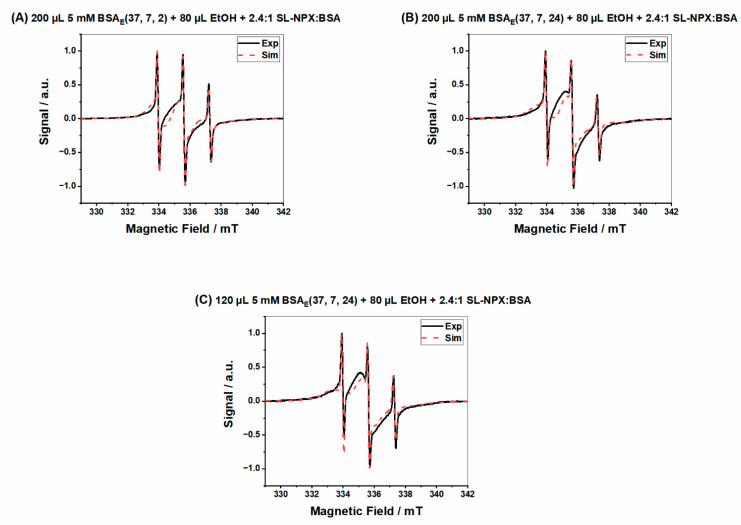
(**A**) SL-NPX-loaded BSA_E_(37, 7, 2) at a 2.4:1 SL-NPX:BSA molar ratio with 80 µL ethanol, (**B**) SL-NPX-loaded BSA_E_(37, 7, 24) at a 2.4:1 SL-NPX:BSA molar ratio with 80 µL ethanol, and (**C**) SL-NPX-loaded BSA_E_(37, 7, 24) at a 2.4:1 SL-NPX:BSA molar ratio with 80 µL ethanol.

The direct effect of changing the incubation time and protein concentration on the binding capacities of hydrogels is shown in Figure 6. According to the parameters gained from EPR spectral simulation (see Table 2), we found that increasing the incubation time from 2 to 24 h and the amount of BSA from 120 µL to 200 µL resulted in a higher percentage of strongly bound components in the hydrogel. It seems that a slightly higher fraction of SL-NPX is tightly bound to the binding sites of BSA when hydrogels are prepared by a higher protein concentration and longer incubation times in the thermomixer. These results indicate that ethanol-induced hydrogels can be tuned by changing such parameters and have the potential to be used for drug delivery applications.

#### 2.3.2. SL-NPX Release Studies from BSA Hydrogels

**Figure 7:**
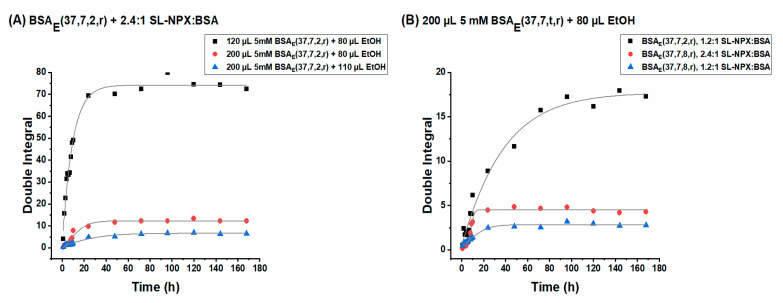
Double integral values as a function of release time intervals for (**A**) SL-NPX-loaded BSA_E_(37, 7, 2, r) at 2.4:1 SL-NPX:BSA molar ratio prepared by different amounts of BSA and ethanol and (**B**) 200 µL 5 mM BSA_E_(37, 7, t, r) prepared by 80 µL ethanol and different SL-NPX:BSA molar ratio.

Following the drug release from different hydrogels allows evaluation of the effect of: (i) ethanol, BSA, and SL-NPX concentrations and (ii) incubation time on the sustained release behavior of SL-NPX. Figure 7 displays the SL-NPX release profile from BSA hydrogels achieved by plotting the double integral of the EPR spectra of the release medium against different time intervals.

With the same initial drug loading amount, the highest release rate is attributed to the hydrogel prepared by 120 µL 5 mM BSA and 80 µL ethanol (see Figure 7A). According to rheological measurements and ATR spectroscopy, a lower concentration of ethanol and BSA leads to mechanically weak hydrogels, which allow faster diffusion of the drug from the gel into the release medium. However, increasing the amount of BSA precursor solution to 200 µL and that of the ethanol to 110 µL results in a significant decrease in drug release. The importance of ethanol concentration on the release behavior can be observed in Figure 7A by comparing the samples prepared with the same amount of BSA and SL-NPX but with different ethanol volumes. It is evident that a lower amount of ethanol leads to a faster and higher release rate.

The effect of initial drug loading and incubation time on the drug release behavior of the BSA hydrogels is shown in Figure 7B. The comparison of the hydrogels prepared by the same incubation time but different SL-NPX concentrations shows that hydrogels containing twice the amount of SL-NPX (2.4:1 SL-NPX:BSA) lead to an increase in the release rate. On the other hand, changing the incubation time from 2 to 8 h for the hydrogels prepared with the same amount of BSA, ethanol, and SL-NPX lowers the release rate due to the formation of a more robust hydrogel as identified by its IR-spectroscopic profile. It is important to mention that for all samples the release rate during the first 24 h is greater than during later time periods and was followed by sustained release over time. The initial release of a huge amount of SL-NPX is due to the fast dissolution of entrapped drugs close to or at the surface of BSA hydrogels by diffusion of release medium into the protein matrix. The later sustained release is attributed to the slow penetration of PBS medium and increased length of the diffusion process as a result of drug depletion in the inner part of the protein matrix. Therefore, the release rate can be altered by changing the mentioned parameters, allowing the use of BSA hydrogels in controlled drug delivery applications. The effect of mentioned parameters on the release behavior for hydrogels formed by different amounts of BSA, ethanol, and SL-NPX as well as other incubation times is described in more detail in the Supporting Information.

**Figure 8:**
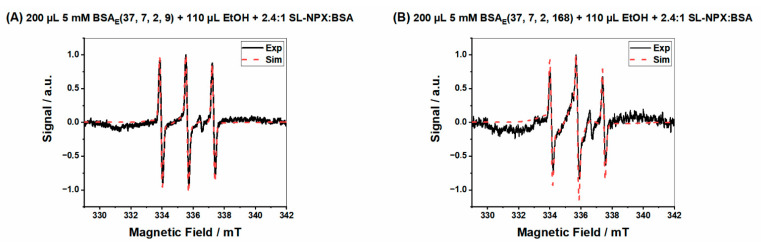
EPR spectra of release test for hydrogels prepared by 200 µL 5 mM BSA, 110 µL ethanol and 2.4:1 SL-NPX:BSA molar ratio of (**A**) BSA_E_(37, 7, 2, 9) and (**B**) BSA_E_(37, 7, 2, 168).

Figure 8A,B shows examples of EPR spectra gained by analyzing the release medium after 9 h and 168 h for ethanol-induced hydrogels prepared by the same amount of BSA (200 µL) and ethanol (110 µL) and SL-NPX:BSA molar ratio (2.4:1) and 2 h of incubation time.

**Figure 9:**
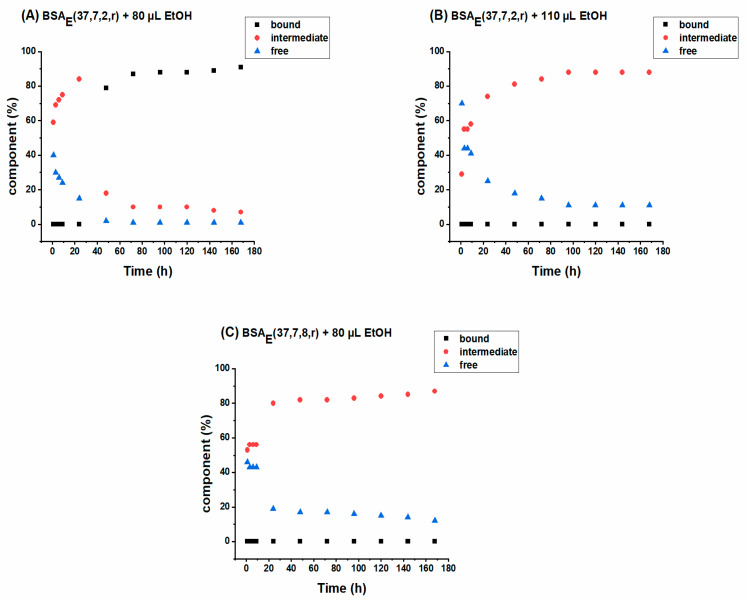
The released percentage of freely rotating SL-NPX, intermediately and tightly bound SL-NPX to BSA versus time intervals of (**A**) BSA_E_(37, 7, 2, r) with 80 µL ethanol, (**B**) BSA_E_(37, 7, 2, r) with 110 µL ethanol, and (**C**) BSA_E_(37, 7, 8, r) with 80 µL ethanol. In these samples, the molar ratio of SL-NPX:BSA is 1.2:1.

The relative fractions of tightly and intermediately bound SL-drug and freely tumbling SL-NPX in the release medium were obtained by spectral simulation which provides more molecular insight into the released structures. The interaction of SL-NPX with BSA during the release test for the samples prepared by 200 µL 5 mM BSA and 1.2:1 SL-NPX:BSA molar ratio is shown in Figure 9. As can be seen in Figure 9A, almost 80% of released SL-NPX is attached intermediately to BSA, 20% tumbles freely, and no strongly bound component is found within the first 24 h of the release (hydrogel from 80 µL ethanol and 2 h of incubation time). However, the percentage of strongly bound SL-NPX to BSA increased over longer time periods later, and those of the intermediately bound and freely rotating decreased sharply. As it was explained in the rheology section, mechanically loose hydrogels can be obtained by the addition of small volumes of ethanol. Therefore, weaker gel structures allow the release of full BSA molecules (or aggregates thereof), in which SL-NPX are transported. In contrast, by increasing the amount of ethanol to 110 µL (Figure 9B) and the incubation time to 8 h (Figure 9C), the fraction of intermediately bound SL-NPX increases over release time, and that of the freely rotating SL-NPX decreases. Moreover, for both samples, there is no sign of strongly bound SL-NPX. This is due to higher ethanol concentrations and incubation times resulting in more robust α-sheet-based hydrogel structures; therefore, less individual (α-helix-containing) albumin molecules and albumin aggregates can be released.

### 2.4. Investigation of Released Components by DLS

**Figure 10:**
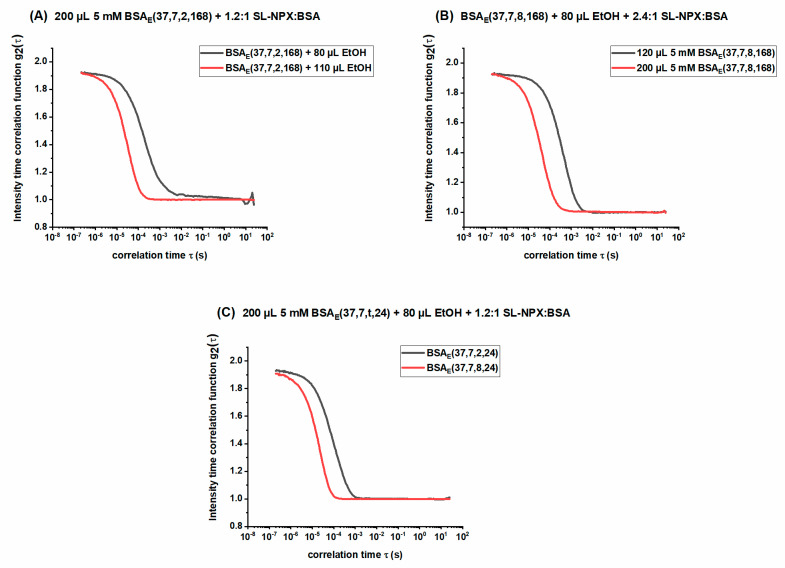
Intensity-time correlation functions for (**A**) SL-NPX-loaded BSA_E_(37, 7, 2, 168) at a 1.2:1 SL-NPX:BSA molar ratio prepared with different amounts of ethanol, (**B**) SL-NPX-loaded BSA_E_(37, 7, 8, 168) at a 2.4:1 SL-NPX:BSA molar ratio prepared with 80 µL ethanol and different BSA concentrations, and (**C**) SL-NPX-loaded BSA_E_(37, 7, t, 24) at a 1.2:1 SL-NPX:BSA molar ratio prepared with 80 µL ethanol.

Dynamic light scattering (DLS) is employed to monitor the hydrodynamic radius and intensity–time correlation function, which provide information on the size of the released components over time. The autocorrelation functions of the release medium obtained from DLS analysis are displayed in Figure 10. As shown in this figure, all recorded correlation functions show pronounced y-intercept values and high scattering intensities, implying the existence of highly defined particles in the PBS medium. Figure 10A shows autocorrelation functions of the release medium after 168 h for ethanol-induced hydrogels (incubation for 2 h and prepared by 200 µL 5 mM BSA) made with two different amounts of ethanol; therefore, it is possible to clearly see the effect of ethanol concentrations on the size of the released particles. We find that the sample with a lower amount of ethanol displays slower decay, indicating the release of larger structures. These results are in line with those of the rheological measurements and CW EPR spectroscopy as hydrogels prepared with lower ethanol volumes are mechanically weak, which leads to the release of larger albumin molecular aggregates formed during gelation.

Figure 10B,C shows the direct effect of BSA concentration and incubation time on the decay of intensity time correlation functions, respectively. As can be seen in Figure 10B, both samples were prepared with 8 h of incubation time, with the same amount of ethanol and SL-NPX:BSA molar ratio, but different amounts of BSA. The sample with 200 µL BSA decayed much faster, which is attributed to the release of smaller particles. Figure 10C displays the same results, as the autocorrelation of the sample prepared by a longer incubation time (8 h) shows fast decay. In the other sections, we have explained in detail that higher concentrations of BSA and longer incubation times can result in mechanically robust hydrogels. Moreover, according to Figure 9, there is no SL-NPX strongly immobilized to BSA when the hydrogels were synthesized by a high amount of ethanol or long incubation time. Therefore, the absence of tightly bound SL-pharmaceuticals, which are larger components than intermediately and freely rotating ones, leads to the faster decay of the autocorrelation function. Further information about the effect of the mentioned parameters on decay rate during other release times can be found in Supporting Information.

## 3. Materials and Methods

### 3.1. Materials

Fatty acid-free bovine serum albumin (lyophilized powder, >96%) was purchased from Sigma-Aldrich (Burlington, MA, USA). All chemicals and materials were used as received. 4-hydroxy TEMPO-labeled naproxen was synthesized previously [34]. For a better understanding and description of parameters analyzed in the research, we have previously [15] introduced a concise notation as follows: BSAi (Θ, p, t, r), where i indicates the gelation technique, which can be thermally (T), pH (P), or the newly established ethanol-induced (E) method, Θ and p specify temperature and pH of gel formation, t and r denote incubation time (in hours) and time-release (in hours), which refers to the release of NPX over a specific period of time. For instance, BSAE (37, 7, 9, 72) describes a hydrogel processed at 37 °C and pH 7 by keeping the sample for 9 h in a thermomixer. Moreover, the release behavior of this gel was studied 72 h after hydrogel formation.

### 3.2. Methods

BSA powder was dissolved in deionized water under constant stirring at 100 rpm for 1 h at room temperature to form 5 mM or 3 mM BSA stock solutions, respectively. A 0.45 µm nylon filter was used to filtrate the obtained solution. SL-NPX was dissolved in DMSO and added to the BSA precursor solution with different BSA and drug ratios. Afterwards, various amounts of ethanol were added to the mixture of BSA and SL-NPX, and the desired final volume was reached by the addition of Milli-Q water. The final mixed solutions were kept in a thermomixer at 37 °C with different incubation times to achieve the final gel.

In vitro drug release from the hydrogels was studied by the addition of 1 mL 10 × PBS (phosphate-buffered saline, pH 7.4) on top of the prepared hydrogels while keeping vials in a thermomixer at 37 °C. At specific time intervals, some amount of release medium was taken out and analyzed with CW EPR and DLS and the same amount of fresh PBS was added back to the medium in order to maintain sink conditions.

### 3.3. Rheological Characterization

To study the mechanical properties as well as the viscoelastic behavior of the hydrogels, the storage (G′) and loss (G′′) moduli are measured by time-dependent rheological characterization. The gelation point presented herein is defined as the time when G′ starts to deviate from G′′ sharply, i.e., when the elastic behavior of the gel predominates the viscous properties. Furthermore, more information on the robustness and stability of hydrogels can be obtained by evaluating the shape and magnitude of storage and loss modulus during gel formation.

For this experiment, a Physica MCR 301 rheometer (Anton Paar, Graz, Austria) equipped with a CP50-2/TG plate as a measurement system was used. By placing 2000 µL of liquid samples on the surface of the rheometer plate, covering the measurement gap with silicon oil to avoid water evaporation, and lowering the closing gap, the storage and loss moduli were recorded. Oscillatory strain and frequency of oscillation were set to 0.5% and 1 rad s^−1^, respectively.

### 3.4. ATR IR Spectroscopy

The application of attenuated total reflection (ATR) Fourier transform infrared spectroscopy (FTIR) for the characterization of protein secondary structures content is based on the assessment of amide I and amide II bands. Stretching vibrations of C=O bonds correspond to the amide I band which is found between 1600 and 1700 cm^−1^, while the amide II band occurs at 1500–1600 cm^−1^ and mainly arises from the bending vibration of N-H bonds [14,47].

ATR IR spectroscopy was used to investigate how different ethanol concentrations cause changes in the secondary structure of BSA during hydrogel formation. A Bruker Tensor 27 FT-IR spectrometer was used that was equipped with a BioATRCell II and an LN-MCT photovoltaic detector and the OPUS Data Collection Program (all from Bruker, Ettlingen, Germany). Thirty microliters of the precursor solution was placed onto the Si-crystal before increasing the temperature using a circulation water bath (HAAKE C25P thermostat). Data acquisition was started when the desired incubation temperature was reached. For each sample, 256 scans at 4 cm^−1^ resolution within the range between 4000 and 900 cm^−1^ were recorded. All spectra were acquired in a dry atmosphere to eliminate pollution. The empty IR cell was used as a reference. The water and ethanol contributions need to be removed before further analysis by subtraction of these spectra recorded under the same condition.

### 3.5. Continuous Wave Electron Paramagnetic Resonance (CW EPR) Spectroscopy

Electron paramagnetic resonance (EPR) spectroscopy is a noninvasive, sensitive, and highly selective technique that can be used to gain information on the electronic structures as well as the local environment of paramagnetic centers with unpaired electrons. In most materials science and biological systems, with the exception of transition metal ions and transient radicals, free electrons have to be incorporated into the desired system by spin labeling or spin probing techniques, which require the covalent or non-covalent attachment of paramagnetic substances into the drug, protein or both [48,49,50]. For the CW EPR investigation of drug–protein interactions in drug delivery systems, organic nitroxide radicals as paramagnetic tracer molecules have been used extensively [14,15]. In this work, information on the release mechanisms and profiles is obtained by plotting the double integral of the EPR spectra against release time. Moreover, by analyzing the changes in the rotational mobility of the spin probes, it is possible to discriminate those SL-drugs tightly attached to BSA from those tumbling freely in the water-swollen region in the hydrogel and the release medium.

All EPR spectra were recorded at X-band frequencies of 9.4 GHz using a Miniscope MS400 (Magnettech, Berlin, Germany, now Bruker Biospin, Ettlingen, Germany) benchtop spectrometer. The temperature was adjusted to 37 °C for all experiments with a temperature controller H03 (Magnettech). A microwave power of 15 mW, sweep width of 15 mT, modulation amplitude of 0.2 or 0.02 mT (for analyzing release medium and hydrogels, respectively), and 4096 points were used during the measurements. Recorded EPR spectra were simulated in MATLAB using the program package EasySpin which is based on applying the Schneider–Freed model to solve the Schrödinger equation for slow rotation nitroxide EPR spectra [23]. The samples were analyzed directly after filling 12 µL of the desired solution into a 50 µL glass capillary (Blaubrand, Wertheim, Germany), which was capped with tube sealant (Leica Critoseal, Wetzlar, Germany).

### 3.6. Dynamic Light Scattering

Dynamic light scattering (DLS) is a relatively fast technique particularly used for measuring the size of small molecules in a liquid environment and studying nanoparticle aggregation [51]. DLS detects time-dependent fluctuations of scattered intensity caused by the Brownian motion of the particle [52]. The particle’s translational diffusion coefficient and hence its hydrodynamic radius can be obtained by analyzing the decay rate of the correlation function [53].

DLS data were determined using a Litesizer 500 (Anton Paar GmbH, Graz, Austria). A 40 nm semiconductor laser with a wavelength of 658 nm was used to illuminate the sample solution. We used 6 runs with an individual duration of 30 s for measurement of each sample and the temperature was adjusted to 37 °C with 2 min of equilibration time. To obtain the hydrodynamic radius and intensity correlation function, 100 µL of release medium was transferred into a quartz cuvette (Hellma Analytics, Mullheim, Germany) without further filtration and measured at a 90° angle (side scattering). Data analysis was achieved with a narrow analysis mode using the mass-averaged values.

## 4. Conclusions

In this paper, ethanol-induced hydrogels have been developed and characterized, with a special focus on use as delivery hosts for controlled and potentially local drug delivery applications. The whole gelation procedure from BSA at 37 °C by the addition of various amounts of ethanol was established in detail and the mechanical properties, as well as changes in the secondary structure of protein, were studied. The application of these hydrogels as suitable carriers and release vehicles was studied by loading different ratios of SL-NPX into these structures. We have compared the viscoelastic behavior and drug–protein interaction of the ethanol-induced hydrogels with other gelation methods, namely thermally and pH-induced techniques, which were established previously. In particular, for ethanol-induced hydrogels, the drug release features obtained from double integration of CW EPR spectra principally depend on initial drug loading percentage, hydrogel incubation time as well as BSA and ethanol concentrations. Generally, higher drug ratios, shorter incubation times, and lower BSA and ethanol concentrations result in a higher release rate. However, there is a lower limit ethanol concentration, below which the gel network cannot form. The release of the drug in the medium suggests an initial burst release followed by a sustained release during later time periods.

Moreover, CW EPR spectroscopy and DLS measurements provide deeper insight into the interaction of SL-pharmaceutical with hydrogels and the size and nature of released components. We have shown that less robust hydrogels released a higher percentage of SL-NPX tightly bound to BSA than in mechanically strong gels, in which a higher percentage of SL-NPX attached intermediately to BSA hydrogels exists in the release medium. This can be correlated to faster diffusion of water into the looser hydrogel network at low ethanol contents, as then more unreacted albumins as monomers, dimers, or protein aggregates are released from these hydrogels. All of the results indicate the possibility to tailor-make BSA hydrogels by the addition of ethanol that can be used in controlled and sustained drug delivery applications for local drug administration.

## Figures and Tables

**Table 1:** Parameters of spectral simulation of Figure 5.

Figure	Type of Component	Weight Percentage	Correlation Time τc [ns]	Hyperfine Coupling Constant *a*_iso_ [MHz]
**Figure 5C**	Bound	0	0	0
	Intermediate	51%	2	44.5
	Free	48%	0.11	45.8
**Figure 5D**	Bound	22%	18	46.1
	Intermediate	67%	2	45.2
	Free	10%	0.26	46.5
**Figure 5E**	Bound	73%	18	46.4
	Intermediate	24%	3	48.2
	Free	2%	0.45	47.2
**Figure 5F**	Bound	65%	28	42.2
	Intermediate	31%	2	44.2
	Free	4%	0.36	46.5

**Table 2:** Parameters of spectral simulation of Figure 6.

Figure	Type of Component	Weight Percentage	Correlation Time τ_c_ [ns]	Hyperfine Coupling Constant *a*_iso_ [MHz]
**Figure 6A**	Bound	7%	14	46.1
	Intermediate	86%	2.1	46.5
	Free	6%	0.26	46.5
**Figure 6B**	Bound	12%	14	46.1
	Intermediate	86%	3.8	45.5
	Free	2%	0.56	46.5
**Figure 6C**	Bound	4%	15	44.7
	Intermediate	91%	8.6	46.5
	Free	5%	0.42	46.5

## Data Availability

Data are available from the authors upon request.

## Supplementary Materials

The following supporting information can be downloaded at: https://www.mdpi.com/article/10.3390/ijms23137352/s1.
