# Supplementary material for: Macro- and Nanoscale Effect of Ethanol on Bovine Serum Albumin Gelation and Naproxen Release
Source: Int J Mol Sci. 2022 Jul 1;23(13):7352. doi: 10.3390/ijms23137352 (PMC9266526; doi:10.3390/ijms23137352)
Supplement: Supplementary file 1 [file ijms-23-07352-s001.zip › ijms-1761908-supplementary.pdf]

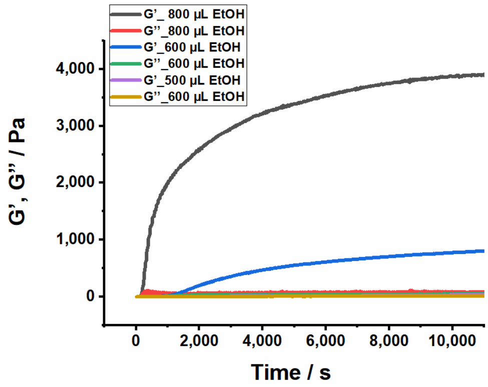


Figure S1. Time-dependent elastic (G′) and viscous moduli (G′′) of 1000 µL 3 mM $BSA_E$(37, 7) with different amounts of ethanol.

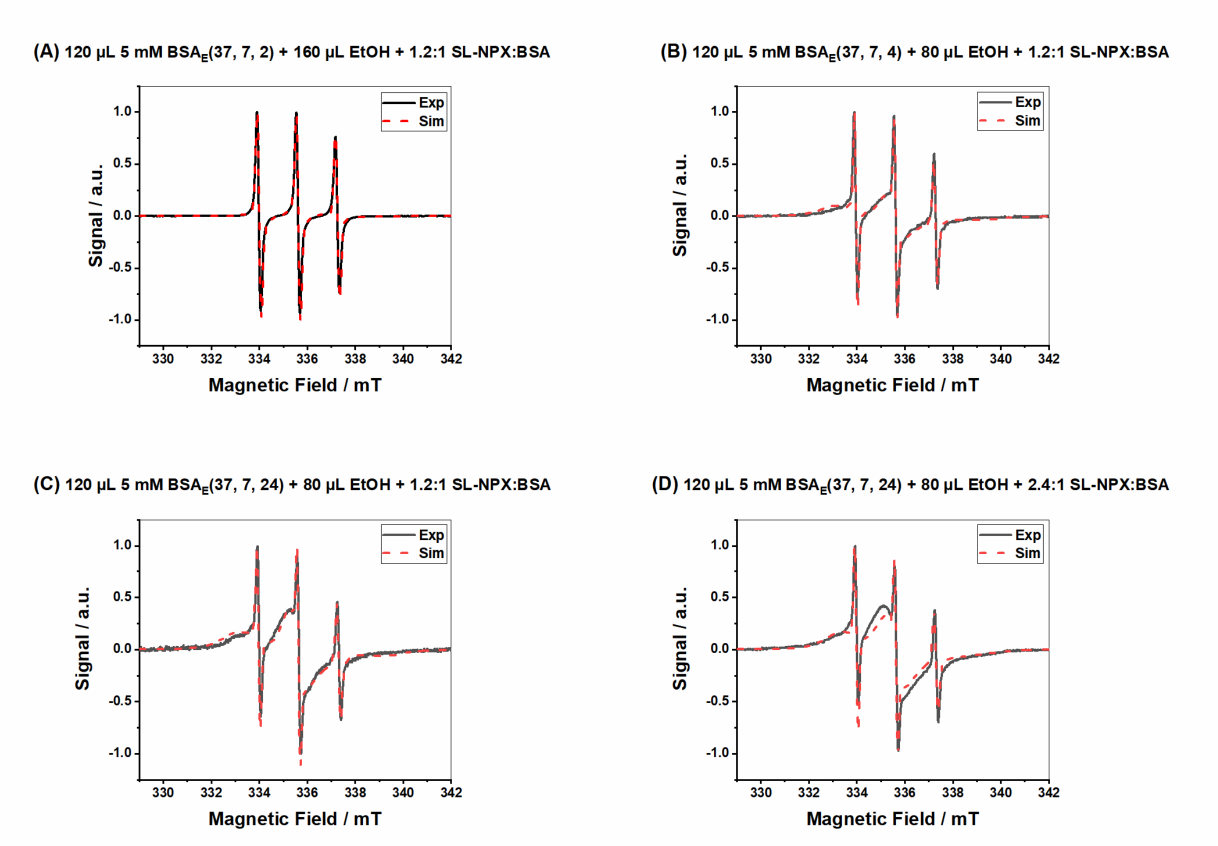


Figure S2. (A) SL-NPX loaded $BSA_E$(37, 7, 2) at a 1.2:1 SL-NPX: BSA molar ratio with 160 μL ethanol, (B) SL-NPX loaded $BSA_E$(37, 7, 4) at a 1.2:1 SL-NPX: BSA molar ratio with 80 μL ethanol, (C) SL-NPX loaded $BSA_E$(37, 7, 24) at a 1.2:1 SL-NPX: BSA molar ratio with 80 μL ethanol and (D SL-NPX loaded $BSA_E$(37, 7, 24) at a 2.4:1 SL-NPX: BSA molar ratio with 80 μL ethanol.

Table S1. Parameters of spectral simulation of Figure S2.

| Figure | Type of components | Percentage | Correlation time τc [ns] | Hyperfine coupling constant aiso [MHz] |
|---|---|---|---|---|
| | Bound | 0 | 0 | 0 |
| A | Intermediate | 51% | 2 | 44.5 |
| | Free | 48% | 0.11 | 45.8 |
| | Bound | 0 | 14 | 46.1 |
| B | Intermediate | 94% | 9 | 47.5 |
| | Free | 6% | 0.1 | 46.5 |
| | Bound | 2% | 13 | 47.1 |
| C | Intermediate | 95% | 4 | 46.2 |
| | Free | 3% | 0.47 | 46.8 |
| | Bound | 5% | 15 | 44.7 |
| D | Intermediate | 91% | 8 | 46.5 |
| | Free | 3% | 0.46 | 46.5 |

By comparing the parameters obtained from EPR spectral simulation of figure S2A with S2B we can realize that increasing the amount of ethanol leads to lower percentage of freely tumbling SL-NPX. Moreover, increasing the incubation time from 4 hours to 24 hours (see Figure S2C and S2D) results in the higher percentage of strongly bound SL-NPX to BSA in the hydrogel.

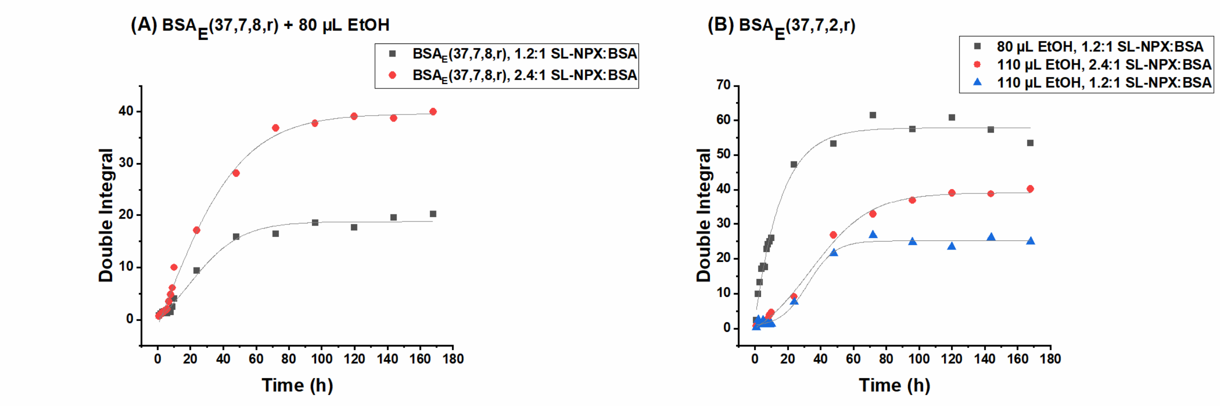


Figure S3. Double integral values as a function of release time intervals for (A) SL-NPX loaded BSA$_E$(37, 7, 8, r) prepared by addition of 80 µL ethanol and different molar ratios of SL-NPX: BSA and (B) BSA$_E$(37, 7, 2, r) prepared by addition of different ethanol amounts and different SL-NPX: BSA molar ratio. All of the samples are prepared with 120 µL BSA 5 mM.

Figure S3A shows the direct effect of initial drug loading on release rate, as higher molar ratios of SL-NPX can lead to larger double integral value, i.e. higher release rate. As can be seen in Figure S3B, increasing the amount of ethanol from 80 µL to 110 µL results in lower release rate due to stronger mechanical properties. In rheological characterizations section, we have completely explain the effect of ethanol on viscoelastic behavior of the BSA hydrogels.

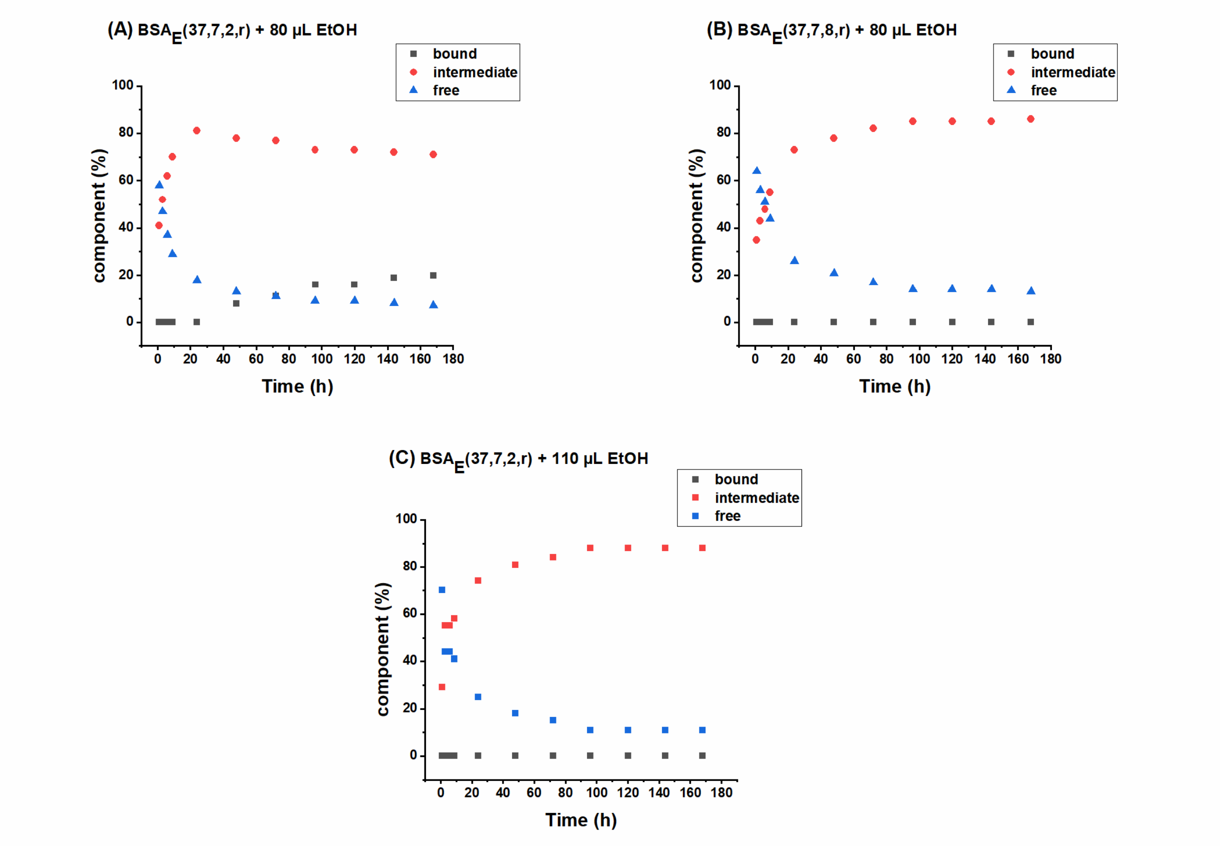


Figure S4. The released percentage of freely rotating SL-NPX, intermediately and tightly bound SL-NPX to BSA versus time intervals of (A) $BSA_E$(37, 7, 2, r) with 80 µL ethanol at a 2.4:1 SL-NPX: BSA molar ratio, (B) $BSA_E$(37, 7, 8, r) with 80 µL ethanol at a 2.4:1 SL-NPX: BSA molar ratio and (C) $BSA_E$(37, 7, 2, r) with 110 µL ethanol at a 1.2:1 SL-NPX: BSA molar ratio. In these samples the precursor solutions are prepared with addition of 200 µL BSA 5 mM.

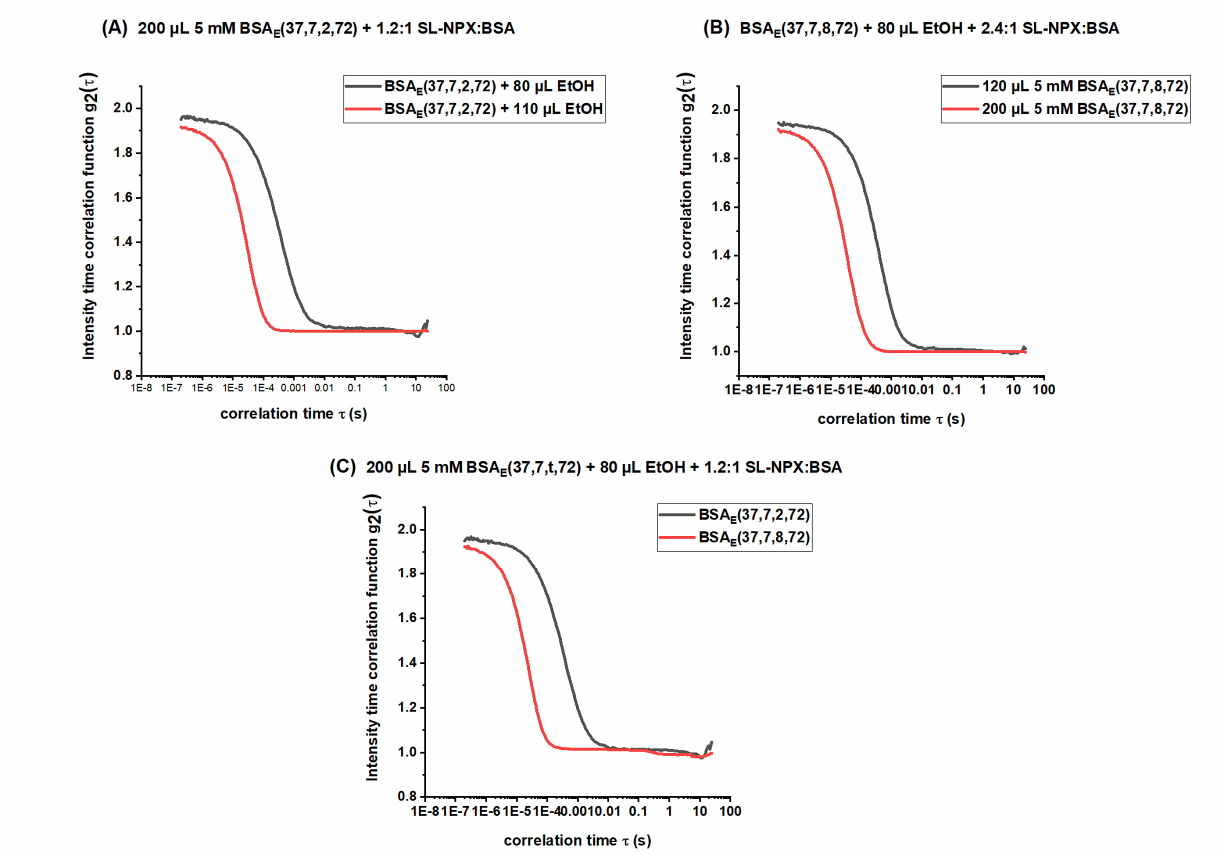


Figure S5. Intensity time correlation functions for (A) SL-NPX loaded $BSA_E$(37, 7, 2, 72) at a 1.2:1 SL-NPX: BSA molar ratio prepared with different amounts of ethanol, (B) SL-NPX loaded $BSA_E$(37, 7, 8, 72) at a 2.4:1 SL-NPX: BSA molar ratio prepared with 80 µL ethanol and different BSA concentrations and (C) SL-NPX loaded $BSA_E$(37, 7, t, 72) at a 1.2:1 SL-NPX: BSA molar ratio prepared with 80 µL ethanol.
